# BUSCO Applications from Quality Assessments to Gene Prediction and Phylogenomics

**DOI:** 10.1093/molbev/msx319

**Published:** 2017-12-06

**Authors:** Robert M Waterhouse, Mathieu Seppey, Felipe A Simão, Mosè Manni, Panagiotis Ioannidis, Guennadi Klioutchnikov, Evgenia V Kriventseva, Evgeny M Zdobnov

**Affiliations:** Department of Genetic Medicine and Development, University of Geneva Medical School and Swiss Institute of Bioinformatics, Geneva, Switzerland

**Keywords:** transcriptomics, metagenomics, bioinformatics, evolution

## Abstract

Genomics promises comprehensive surveying of genomes and metagenomes, but rapidly changing technologies and expanding data volumes make evaluation of completeness a challenging task. Technical sequencing quality metrics can be complemented by quantifying completeness of genomic data sets in terms of the expected gene content of Benchmarking Universal Single-Copy Orthologs (BUSCO, http://busco.ezlab.org). The latest software release implements a complete refactoring of the code to make it more flexible and extendable to facilitate high-throughput assessments. The original six lineage assessment data sets have been updated with improved species sampling, 34 new subsets have been built for vertebrates, arthropods, fungi, and prokaryotes that greatly enhance resolution, and data sets are now also available for nematodes, protists, and plants. Here, we present BUSCO v3 with example analyses that highlight the wide-ranging utility of BUSCO assessments, which extend beyond quality control of genomics data sets to applications in comparative genomics analyses, gene predictor training, metagenomics, and phylogenomics.

Genomics approaches play a preeminent role in biological research because they are high-throughput and cost-effective, leading to the generation of ever-increasing volumes of data. This makes thorough quality control of sequencing data “products”, for example, genomes, genes, or transcriptomes, ever more important. Addressing this, the Benchmarking Universal Single-Copy Ortholog (BUSCO, http://busco.ezlab.org) assessment tool provides intuitive quantitative measures of genomic data completeness in terms of expected gene content (Simão et al. 2015). BUSCO identifies complete, duplicated, fragmented, and missing genes and enables like-for-like quality comparisons of different data sets. These features mean that BUSCO has become established as an essential genomics tool, using up-to-date data from many species and with broader utilities than the popular but now discontinued Core Eukaryotic Genes Mapping Approach (CEGMA) (Parra et al. 2007). In this communication, we present the major BUSCO improvements, now in its third release as detailed below, with scenarios that highlight BUSCO’s wide-ranging genomics utilities: designed primarily for performing genomics data quality control, but also applicable for building robust training sets for gene predictors, selecting high-quality reference species for comparative genomics analyses, and identifying reliable markers for large-scale phylogenomics and metagenomics studies.

## New Approaches

### BUSCO v3: Enhanced Features and Extended Data Sets

Since the initial BUSCO release, development has aimed to address user needs with BUSCO v2 implementing improvements to the underlying analysis software as well as updated and extended data sets covering additional lineages based on orthologs from OrthoDB v9 (Zdobnov et al. 2017). For example, as well as the bacteria-wide data set, there are now 15 additional lineage-specific data sets, and the fungal data sets additionally comprise nine lineage-specific data sets while Metazoa is now made up of 12 subsets including vertebrates and arthropods, and additional data sets have been built for nematodes, plants, and protists. To facilitate high-throughput assessments, BUSCO v3 now implements a refactoring of the code to make it more flexible and extendable by simplifying installation and introducing control through a configuration file. Additionally, visualization of the results is enabled with a plotting tool that generates easily configurable bar charts. The software is distributed through GitLab, it is now also available as an Ubuntu virtual machine, and it has been integrated as an online service for logged-in users at www.orthodb.org. These and other new features, options, software setup instructions, dependencies including BLAST + (Camacho et al. 2009) for sequence searches, HMMER (Eddy 2011) hidden Markov models (HMMs) for profile searches, and Augustus (Keller et al. 2011) for block-profile-based gene prediction, as well as best practices are all described in detail in the updated user guide (http://busco.ezlab.org). With many more new species being sequenced, future BUSCO releases will focus on adding new lineages for which species sampling becomes rich enough to build reliable data sets as well as providing higher resolution with larger lineage-specific data sets.

## Results

### Assessing Genome, Gene Set, and Transcriptome Completeness

**Fig. 1:**
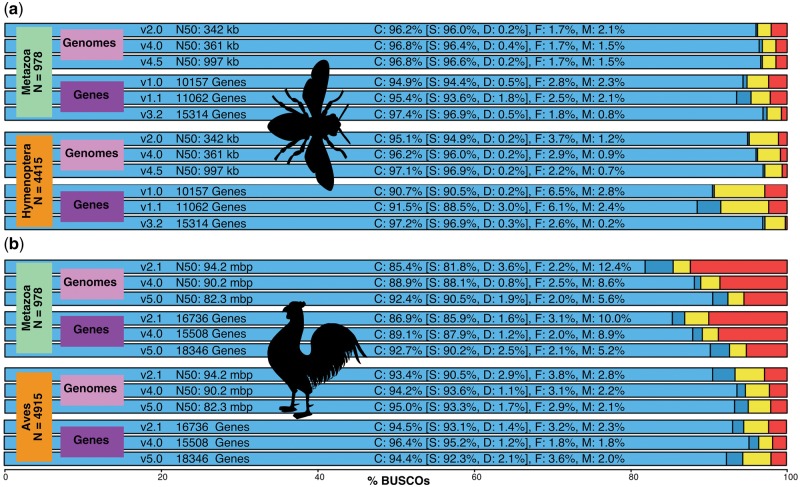
BUSCO completeness assessments for genomics data quality control. Assessments of initial, intermediate, and latest versions of the (*a*) honeybee and (*b*) chicken genomes and their annotated gene sets with the Metazoa, Hymenoptera, and Aves lineage data sets. Bar charts produced with the BUSCO plotting tool show proportions classified as complete (C, blues), complete single-copy (S, light blue), complete duplicated (D, dark blue), fragmented (F, yellow), and missing (M, red).

Genomics data quality control motivated the delineation of the original BUSCO data sets (Waterhouse et al. 2013) and their subsequent integration with the assessment tool for analyzing the completeness of genome assemblies, annotated genes, and transcriptomes (Simão et al. 2015). Benchmarking new genomes or gene sets against those of gold-standard model organisms or of closely related species provides intuitive like-for-like comparisons. For transcriptomes, high completeness is expected for samples pooled from multiple life stages and tissues, whereas lower scores for targeted samples corroborate their specificity. Benchmarking can also help to guide iterative reassemblies or reannotations toward quantifiable improvements, for example, the postman butterfly (Davey et al. 2016) and Atlantic cod (Tørresen et al. 2017). Here, we assess three versions of the annotated chicken and honeybee genomes (Materials and Methods), which have been the subject of extensive enhancements (Elsik et al. 2014; Warren et al. 2017) and clearly demonstrate the utility of BUSCO for quantifying successful improvements (fig. 1). Progressions from the initial, to intermediate, and latest versions of both species show improved completeness using the high-resolution Hymenoptera or Aves data sets and the lower resolution Metazoa data set.

### High-Quality Training Data Sets for Improved Gene Prediction

**Fig. 2:**
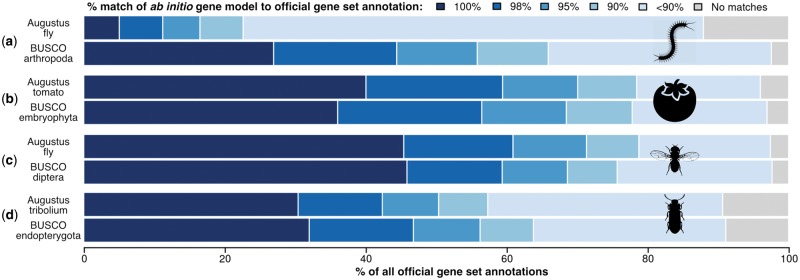
BUSCO-trained ab initio gene prediction with Augustus. When no pretrained parameter set is available, for example, for (*a*) the centipede, BUSCO-trained predictions are substantially better than using Augustus parameters from another arthropod (fly). Where species-specific-trained parameter sets are available, BUSCO-trained predictions are almost as good, for example, (*b*) tomato, just as good, for example, (*c*) fruit fly, or even better, for example, (*d*) *Tribolium* beetle. Performance was assessed by computing the percent sequence length match of the ab initio gene models to the official gene set annotations for each species (Materials and Methods).

Gene predictor training exemplifies BUSCO utilities beyond quality control, as gene models built during genome assessments represent ideal input data for parameterizations. Accurate prediction of protein-coding genes remains challenging, especially when supporting evidence such as homologs or native transcripts is not available and predictions are performed ab initio. This involves statistical modelling of nucleotide signatures and content to build gene models that best fit pretrained parameter distributions. These vary considerably among species and thus require optimization, often employing high-quality gene annotations from native transcripts as input data. BUSCOs represent complementary predefined sets for such training procedures, without the need to perform RNA sequencing. Comparing Augustus predictions using BUSCO-trained parameters versus available pretrained parameters from other species (Materials and Methods) can show substantial improvements, for example, BUSCO-trained *Strigamia* centipede, *Daphnia* waterflea, and *Danaus* butterfly predictions are much better than using fruit fly parameters (fig. 2 and supplementary fig. S1, Supplementary Material online). Where species-specific-trained parameters are available, BUSCO training performs almost as well, for example, tomato and thale cress, just as well, for example, fruit fly and *Nasonia* wasp, or even better, for example, *Tribolium* beetle (fig. 2 and supplementary fig. S1, Supplementary Material online). Thus even if BUSCO gene models may not include all protein-coding exons, for example, if some divergent exons are not predicted, they provide ample training data (native intron–exon boundaries) to improve ab initio gene finding. BUSCO employs Augustus for gene prediction so assessing genomes automatically generates Augustus-ready parameters trained on genes identified as complete. Additionally, the BUSCO-generated general feature format and GenBank-formatted gene models can be used as inputs for training other gene predictors like SNAP (Korf 2004). Running assembly assessments therefore provides users with high-quality gene model training data that can greatly improve genome annotation procedures.

### Informed Data Set Sampling for Robust Comparative Genomics

Comparative genomics analyses are often sensitive to incomplete data, making the selection of high-quality data sets from representative species a critical first step for many studies. This becomes increasingly complex as the amount of available genomics data grows, especially as quality may vary considerably. Quantifying completeness can help to make objective selections, for example, surveying 653 *Streptomyces* genomes identified the full complement of complete bacteria BUSCOs for only 63% of them (Studholme 2016). Selecting those with the most genes does not guarantee quality, as genomes with many genes are not necessarily the most complete and those with fewer genes are not always less complete (Waterhouse 2015). Selections will undoubtedly be influenced by considerations of taxonomic sampling, the availability of pertinent functional genomics data, the extent and/or accuracy of functional annotations, or simply historical usage. However, all else being equal, quantitative assessments with BUSCO offer logical selection criteria to help focus on the most complete genomic resources available. For example, assessing 135 *Lactobacillus* and 35 *Aspergillus* genomes and comparing these with their contiguity measures and total gene counts (Materials and Methods) shows that RefSeq-designated references are not always the best available representatives (supplementary fig. S2, Supplementary Material online). Comparing such metrics in this way therefore allows for the informed selection of the best quality representatives for subsequent comparative analyses.

### Reliable Marker Selection for Phylogenomics and Metagenomics

**Fig. 3:**
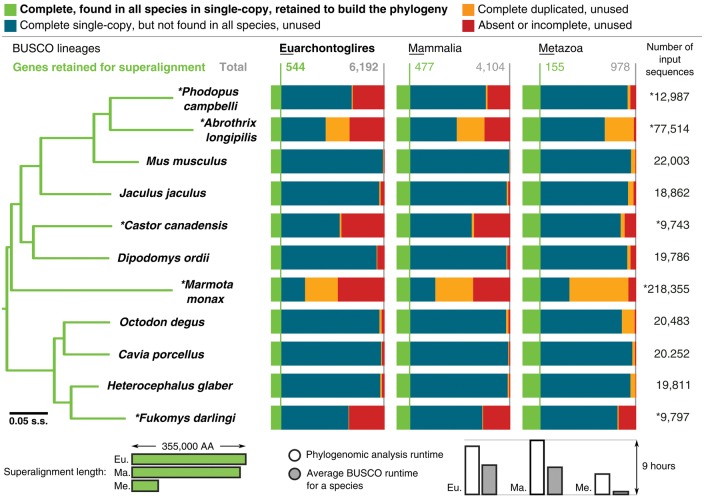
Genome and transcriptome BUSCO assessments to identify universal single-copy markers for phylogenomics studies. The phylogeny was generated using the Euarchontoglires results to identify complete single-copy orthologs found in all species for building the superalignment used for maximum likelihood tree reconstruction (Materials and Methods). Mammalia and Metazoa results produced identical tree topologies. Bars below the BUSCO results show how the sizes of the assessment data sets influence the superalignment lengths and the analysis runtimes. The tree was rooted with the rabbit, all nodes have 100% bootstrap support, branch lengths are in substitutions per site (s.s.).

Phylogenomics takes advantage of whole genome or transcriptome data to reconstruct phylogenies that chart the relationships among organisms, a prerequisite for almost any evolutionary study. Recent notable examples include whole genome sequencing to build a well-supported avian phylogeny (Jarvis et al. 2014) and explore gene flow in mosquitoes (Fontaine et al. 2015), and extensive transcriptomics to increase species sampling to examine the evolution of insects (Misof et al. 2014; Peters et al. 2017) and spiders (Fernández et al. 2014). Being near-universal single-copy genes, BUSCOs represent predefined sets of reliable markers where assessments can identify shared subsets from different types of genomic data. For example, employing BUSCOs from insect genomes and transcriptomes to confirm Odonata–Neoptera relationships (Ioannidis et al. 2017), and from nearly 100 fungal genomes to reconstruct the Saccharomycotina phylogeny (Shen et al. 2016). Analysis of seven rodent genomes and five transcriptomes illustrates the use of BUSCO to recover genes for phylogenetic inference (fig. 3). The identified genes were used to build a superalignment from which to estimate the species phylogeny (Materials and Methods), which agrees with previous studies (Huchon et al. 2007; Blanga-Kanfi et al. 2009). Assessments with the high-resolution Euarchontoglires or Mammalia data sets take longer but they identify more than three times as many universal single-copy markers than the lower resolution Metazoa data set. This illustrates the utility of BUSCO assessments to relatively quickly and easily identify reliable single-copy markers from different types of genomic data for phylogenomics analyses. Universal molecular markers are also essential in metagenomics studies, for phylogenetic classification of the surveyed microbiota, and where estimating relative abundances is greatly simplified if the markers are single-copy (Sunagawa et al. 2013). Hence BUSCOs also represent ideal markers for applications in metagenomics.

## Discussion

BUSCO data sets comprise genes evolving under “single-copy control” (Waterhouse et al. 2011), that is, within each lineage they are near-universally present as single-copy orthologs. While allowing for rare gene duplications or losses, this property underlies the evolutionary expectation that they should be present, and present only once, in a complete assembly or gene set. Completeness is quantified in terms of this expected gene content by assessing the orthology status of predicted genes using BUSCO sequence profiles. These HMM profiles are built from multiple sequence alignments of orthologs and capture the conserved alignable amino acids across the species set (even if some orthologs are incomplete annotations). BUSCOs are carefully selected with finely tuned score and length cut-offs that maximize precision and recall, but as both gene prediction and orthology assignment are challenging tasks, assessments may still fall short of 100% correct classification. For example, some BUSCOs classified as missing could be too divergent or have complex gene structures that render them difficult to locate and predict correctly or even partially, or some BUSCOs classified as duplicated might be heterozygous alleles that the assembly procedure failed to collapse (see Supplementary Material online for further discussion on interpreting BUSCO results). Additionally, while input species selection explicitly avoids oversampling closely related species, the choices must be made from currently available resources that are not phylogenetically evenly distributed. With these caveats in mind, BUSCO offers like-for-like assessments for genomics data quality control, which perform well in qualitative comparisons with alternative measures. For example, metrics based on genome alignments that quantified completeness of ultraconserved elements and protein-coding exons by comparing 20 vertebrates to human (Seemann et al. 2015) showed overall very good agreement with BUSCO results. Furthermore, assessing 12 plants (Veeckman et al. 2016) with BUSCO, CEGMA, core plant Gene Families, and Expressed Sequence Tag mapping also showed good agreement. BUSCO therefore offers reliable measures of completeness that agree with alternative approaches, are applicable to different genomic data types, and offer like-for-like comparisons. This utility extends to additional genomics applications including defining data sets for training gene predictors, facilitating objective selection of representatives for comparative studies, and identifying reliable markers for phylogenomics and metagenomics.

## Materials and Methods

Details of the new and updated lineage data sets as well as the new software developments that make up BUSCO v3 are presented in the Supplementary Material online and in the user guide online at http://busco.ezlab.org. BUSCO has been developed and tested on Linux, the codebase is written for Python and runs with the standard Python packages. BUSCO is licensed and freely distributed under the MIT Licence. The BUSCO v3 source code is available through the GitLab project, https://gitlab.com/ezlab/busco, and built as a virtual machine with dependencies preinstalled.

Versions and accessions of all the genome assemblies, annotated gene sets, or transcriptomes assessed by BUSCO as part of this study are detailed in the Supplementary Material online, along with the settings used for each analysis. The Augustus ab initio gene prediction analyses are described in detail in the Supplementary Material online, to compute the coverage scores the predicted protein sequences were aligned against their respective reference annotations using BLASTp (e.g., a coverage score of 100% means that every amino acid of a reference protein is found in the predicted protein with no insertions, deletions, or substitutions). Details of the preprocessing, BUSCO completeness analyses, and postprocessing of the rodent data sets for the phylogenomics study are all presented in the Supplementary Material online, proteins selected for the superalignment were aligned using MAFFT (Katoh and Standley 2013) and filtered with trimAl (Capella-Gutiérrez et al. 2009), and the maximum likelihood tree was built using RAxML (Stamatakis 2014). 

## Supplementary Material

Supplementary data are available at *Molecular Biology and Evolution* online.

## Supplementary Material

Supplementary Data
