# Supplementary material for: BUSCO Applications from Quality Assessments to Gene Prediction and Phylogenomics
Source: Mol Biol Evol. 2017 Dec 6;35(3):543–8. doi: 10.1093/molbev/msx319 (PMC5850278; doi:10.1093/molbev/msx319)

SUPPLEMENTARY MATERIAL

BUSCO applications from quality assessments to gene prediction and phylogenomics

Robert M. Waterhouse[1,2], Mathieu Seppey[1,3], Felipe A. Simão[1,3], Mosè Manni[1], Panagiotis Ioannidis[1], Guennadi Klioutchnikov[1], Evgenia V. Kriventseva[1] & Evgeny M. Zdobnov[1,*]

[1]Department of Genetic Medicine and Development, University of Geneva Medical School and Swiss Institute of Bioinformatics, Geneva, Switzerland. [2]Present address: Department of Ecology and Evolution, University of Lausanne, Lausanne, Switzerland. [3]These authors contributed equally to this work. *Corresponding author: Evgeny.Zdobnov@unige.ch.

SUPPLEMENTARY TEXTS AND FIGURES

Background 2
BUSCO updates: enhanced features and extended datasets 4
Genomics data quality control 8
Gene predictor training sets 10
Comparative genomic analyses 12
Reliable phylogenomics markers 14
Supplementary References 17
Supplementary Figures 20

Background

The falling costs of DNA and RNA sequencing associated with new technologies mean that genomics-based approaches to addressing biological questions are becoming more widely accessible, a so-called ‘democratization’ of genomics. These sequencing data are subsequently processed by myriad different computational applications into genome or transcriptome assemblies and their annotations. Both the increase in the volume of data and the variable computational approaches to data processing make it increasingly important to be able to gauge the quality of the genomics data being produced. Most technologies and tools provide various metrics to help assess data quality throughout the process, allowing diligent researchers to iteratively tweak workflows and parameters to achieve the best results. However, most of these measures ignore the important complementary question of genomic data quality in terms of gene content completeness.

To address this, Benchmarking Universal Single-Copy Ortholog (BUSCO) assessments were designed to provide quantitative measures of the completeness of genome assemblies, annotated gene sets, and transcriptomes in terms of expected gene content (Simão et al. 2015), http://busco.ezlab.org. BUSCO assessments have been widely adopted by genomics research communities for data quality control procedures. However, applications extend well beyond such procedures and include building robust training sets for gene predictors, selecting high-quality reference strains or species for comparative analyses, and identifying reliable markers for large-scale phylogenomics studies.

This flexible utility of BUSCO stems from the definition of an evolutionary expectation of gene content. That is, genes that make up the BUSCO datasets are selected from OrthoDB (Zdobnov et al. 2017) orthologous groups with single-copy orthologs in the majority of species for each major lineage. While allowing for rare gene duplications or losses, this establishes an evolutionarily informed expectation that these genes should be found as single-copy orthologs in any newly-sequenced genome as they are evolving under ‘single-copy control’ (Waterhouse et al. 2011).

This expectation means that if there are many BUSCOs that cannot be identified in a genome assembly or annotated gene set, it is possible that the sequencing and/or assembly and/or annotation approaches have failed to fully capture the complete expected gene content. Furthermore, the identification of large numbers of duplicated BUSCOs with high sequence identity could indicate that the genome assembly procedure has failed to collapse sequenced haplotypes. In this way, BUSCO provides a biologically meaningful completeness metric for genomics data quality control that complements technical measures like contig or scaffold N50 (a summary measure of assembly contiguity where half the genome is assembled into contigs/scaffolds of length N50 or longer).

BUSCO assessments identify complete, duplicated, fragmented, and missing genes, allowing users to quantitatively compare their data to those from gold-standard model organisms or other closely-related species, or to confirm whether their efforts to improve their assemblies or annotations have been effective. These features, and the ability to assess not just genome assemblies but also transcriptomes and annotated gene sets, are innovations that have been widely welcomed by the genomics community. This means that BUSCO has rapidly become well-established as an essential genomics resource for much more than just genome assembly completeness assessment, using up-to-date data across many different species clades from OrthoDB, and with many more applications than the previously very popular, but now discontinued, Core Eukaryotic Genes Mapping Approach, CEGMA (Parra et al. 2007) (see also: http://www.acgt.me/blog/2015/5/18/goodbye-cegma-hello-busco).

In this study, we present a summary of the latest major BUSCO features along with example analysis scenarios that highlight the wide-ranging utility of BUSCO assessments designed primarily for (i) performing **genomics data quality control**, but also notably applicable for (ii) building **gene predictor training** sets, (iii) choosing reference strains or species for **comparative genomics analyses**, and (iv) selecting reliable **phylogenomics markers**.

BUSCO updates: enhanced features and extended datasets

BUSCO datasets were first defined using orthologs from OrthoDB v7 (Waterhouse et al. 2013) and were subsequently incorporated into the BUSCO assessment tool representing 3,023 near-universally conserved genes for vertebrates, 2,675 for arthropods, 843 for metazoans, 1,438 for fungi, 429 for eukaryotes, and 40 for prokaryotes (Simão et al. 2015). Addressing the growing demands from our users, we subsequently released BUSCO v2 that implemented improvements to the underlying analysis software as well as updated and extended datasets of BUSCOs covering additional lineages based on orthologs from OrthoDB v9 (Zdobnov et al. 2017). The analysis software is distributed through GitLab (http://gitlab.com/ezlab/busco), it is also available as an Ubuntu virtual machine, and it has been integrated as an online service for logged-in users at www.orthodb.org. In response to requests from high-throughput users, we then focused on refactoring the code for the BUSCO v3 release to make it more flexible and extendable. This simplifies the setup and installation procedure and introduces the use of a configuration file that makes BUSCO easier to be integrated in high-throughput pipeline workflows.

The BUSCO assessment tool implements an open-source Python-based software to identify and classify near-universal single-copy ortholog matches from genome assemblies, annotated gene sets, or transcriptomes. It employs BLAST+ (Camacho et al. 2009) for sequence searches, HMMER (Eddy 2011) hidden Markov models for profile searches, and Augustus (Keller et al. 2011) for block-profile-based gene prediction. For genome assembly assessments, candidate genomic regions that could harbour BUSCO matches are first identified with tBLASTn searches using BUSCO consensus sequences. These consensus sequences are derived from hidden Markov model (HMM) profiles built from multiple sequence alignments of orthologs and capture the conserved alignable amino acids across the species set (even if some orthologs are incomplete annotations). This represents a key difference from the CEGMA dataset that contains protein sequences from each of six species, while the BUSCO datasets contain consensus protein sequences and variants, i.e. not the actual protein sequences but representations of the conserved alignable amino acids across the species set. This is designed to reduce any potential species bias as any species being assessed that is closely-related to one of the input species would have an unfair advantage if the input

species sequences themselves were used instead of a consensus. From BUSCO v2 this search is enhanced using variant consensus sequences if the initial searches returned no complete matches.

Gene model prediction is then attempted for each of the identified regions using Augustus with BUSCO block profiles. The protein sequences of these predicted genes, or from an annotated gene set, or the longest open reading frame translations from transcriptomes, are then assessed using HMMER and the lineage-specific BUSCO HMM profiles to classify the matches. The recovered matches are classified as 'complete' (C) if their lengths are within the expectation of the BUSCO profile match lengths. If these are found only once they are classified as 'single-copy' (S), or if found more than once as 'duplicated' (D). The matches that are only partially recovered are classified as 'fragmented' (F), and BUSCO groups for which there are no matches that pass the tests of orthology are classified as 'missing' (M). These metrics can be readily summarized in a simple and intuitive notation that includes the size (i.e. resolution) of the BUSCO dataset (n): e.g. C:89.0%[S:85.8%,D:3.2%],F:6.9%,M:4.1%,n:3023.

BUSCO attempts to provide a quantitative assessment of completeness in terms of expected gene content by simplifying the results into the C / S / D / F / M categories. It should be noted that these labels are simplifications of the most likely scenario, which may nonetheless have alternative interpretations (see the updated user guide for further details http://busco.ezlab.org).

Complete BUSCO matches must score within the expected range of scores and within the expected range of length alignments to the BUSCO HMM profile, determined by the scores and alignment lengths of the input orthologs themselves. Score thresholds have been optimised to identify true orthologs but if the ortholog is not present or is only partially present (highly fragmented) and a high-identity homolog is present then this homolog could be mistakenly identified as the complete BUSCO.

Matches labelled as fragmented score within the range of scores but not within the range of length alignments. This indicates incomplete transcripts or gene models from transcriptome or annotated gene set assessments. For assembly assessments this indicates that the gene is only partially present or that the sequence search and gene

prediction steps failed to produce a full-length gene model. Such fragmented matches are given a ‘second chance’ with a second round of sequence searches and gene predictions with parameters trained on round one complete BUSCOs, but this can still fail to recover the whole gene. Thus some of these fragmented BUSCOs could be complete but are just too divergent or have very complex gene structures, making them very hard to locate and predict in full.

If classified as missing there were no significant matches or the matches scored below the range of scores for the BUSCO HMM profile. For transcriptome or annotated gene set assessments this indicates that these BUSCOs are missing or the transcripts or gene models are so incomplete that they could not even meet the criteria to be considered as fragmented matches. For assemblies this indicates either that these BUSCOs are missing, or that the sequence search step failed to identify any significant matches, or that the gene prediction step failed to produce even a partial gene model that might have been recognised as a fragmented BUSCO. BUSCOs missing after the first round are given a ‘second chance’ with a second round of sequence searches and gene predictions with parameters trained on round one complete BUSCOs, but this can still fail to recover the gene. BUSCOs missing from assemblies could be partially present or even possibly complete but they are just too divergent or have very complex gene structures, making them very hard to locate and predict correctly or even partially.

Duplicated BUSCOs have more than one complete match that satisfies the criteria of scores and alignment lengths. Rare gene duplications can and do happen, and the selection of BUSCOs from orthologous groups with single-copy orthologs in the majority (>90%) of species for each major lineage means that some duplications are expected. A high number of duplicates could suggest the presence of non-collapsed haplotypes (alleles) in a genome assembly, but BUSCO results simply report if multiple complete matches are found and thus further independent analyses by the user would be required in order to determine whether this is the case. This is particularly important if users are relying on BUSCO results to select from a variety of different assemblies produced with different parameters or tools or input data, i.e. duplicates should not be ‘selected against’ simply to achieve a lower BUSCO duplicate score.

BUSCO v2 and v3 implement several improvements to the v1 codebase to make assessments faster as well as to provide more comprehensive information on the progress of the analyses and the reporting of any encountered errors or system warnings. For example, additional steps such as formatting the outputs for each predicted gene now take advantage of multiple threads/cores if this option is specified. New optional arguments allow users to automatically compress large results folders, or to restart failed assessments from the last successfully completed step, e.g. to avoid having to unnecessarily rerun the often time-consuming initial tBLASTn searches. Users may now also pass additional optional arguments to Augustus, e.g. in order to select a non-canonical translation table for the gene predictions. Additionally, visualization of the assessment results is now facilitated with the BUSCO plotting tool that allows users to easily generate configurable bar charts using the ggplot2 R-package (Wickham 2009). These new features and other options are described in detail in the updated user guide (http://busco.ezlab.org).

With the increased species sampling available at OrthoDB, and the requests from many users, BUSCO was extended to include many more assessment datasets representing additional lineages and providing higher resolution through larger lineage-specific BUSCO datasets. For example, as well as the bacteria-wide dataset, BUSCO now includes 15 additional lineage-specific datasets from Actinobacteria to Tenericutes, and the fungal datasets additionally comprise 9 lineage-specific datasets from Ascomycota to Sordariomyceta. Metazoa is now made up of 12 subsets including vertebrates and arthropods, and additional datasets have been built for nematodes, plants, and protists. Lineages were selected based on their taxonomic range and coverage in terms of the numbers of available sequenced and annotated genomes, and future releases will include additional lineages for which species sampling becomes rich enough to build reliable BUSCO assessment datasets.

Genomics data quality control

Using universal single-copy orthologs to benchmark newly-sequenced and assembled genomes against those of gold-standard model organisms provides a comparative estimate of assembly quality in terms of gene content completeness. Similarly, BUSCO assessments can also help to guide iterative genome re-assemblies and/or re-annotations towards quantifiable improvements. For example, comprehensive efforts to improve the initial draft genome assembly of the Postman butterfly, *Heliconius melpomene*, resulted in a 4% increase in the number of complete BUSCOs recovered (Davey et al. 2016). A similar improvement was achieved for the Atlantic cod, *Gadus morhua*, by combining sequencing data from Illumina, 454, and PacBio technologies and using a novel assembly reconciliation method (Tørresen et al. 2017).

Assessing assemblies of the thale cress, *Arabidopsis thaliana*, the grape vine, *Vitis vinifera* cv. Cabernet Sauvignon, and the coral fungus, *Clavicorona pyxidata*, showed that FALCON-based assemblies were substantially more contiguous and complete than alternate short- or long-read approaches, with FALCON-Unzip-based assemblies having respectively 95% (compared to 96% for reference TAIR10), 94% (compared with 13% and 5% for SOAPdenovo assemblies), and 85% (compared with 3% for a SOAPdenovo assembly) complete BUSCOs (Chin et al. 2016).

As more initial draft genome assemblies are revised using additional sequencing and/or physical mapping data, BUSCO offers not only a metric to consider when selecting from the results of different assembly strategies, but also a complementary quantification of the improvements achieved in terms of expected gene content.

Comparing BUSCO results from assessing initial versus later versions of different genome assemblies and their annotated gene sets shows, in most cases, improvements in the number of complete BUSCOs recovered. The results of BUSCO genome and gene set assessments presented in Figure 1 (main text) use the updates to the chicken (Warren et al. 2017) and honeybee (Elsik et al. 2014) genomes and their annotations to quantify the improvements made after substantial efforts involving many different approaches and supporting datasets.

The genome assemblies and gene annotations for chicken, *Gallus gallus*, were retrieved from Ensembl's FTP server and correspond to: GalGal 5.0 (Ensembl release 88, GCA_000002315.3), GalGal 4.0 (Ensembl release 85, GCA_000002315.2) and GalGal 2.1 (Ensembl release 67, GCA_000002315.1). The genome assemblies and annotations for the honeybee, *Apis mellifera*, were retrieved from the Hymenoptera Genome Database, and correspond to: genome Amel 2.0 (GCF_000002195.1) and annotation Amel_ogs_1.0, Amel 4.0 (GCF_000002195.3) and annotation Amel_ogs_1.1, Amel 4.5 (GCF_000002195.4) and annotation Amel_ogs_3.2.

BUSCO v2.0.1 was run on each genome and annotation using default settings and the datasets: metazoa_odb9 (chicken and honeybee), aves_odb9 (chicken only) and hymenoptera_odb9 (honeybee only). These analyses represent the typical use of BUSCO to assess the quality of genomics data in terms of expected gene content, providing quantifications of improvements after substantial complementary efforts to enhance the data quality.

The honeybee assembly N50 values and total gene counts both increase from the initial (here v2.0 assembly and v1.0 annotation) to the latest (here v4.5 assembly and v3.2 annotation) versions, with parallel improvements in BUSCO completeness. The greatest improvement is seen in the latest honeybee official gene set when assessed with the high-resolution Hymenoptera lineage dataset (n=4,415), with completeness improving by more than 5%.

The chicken assembly N50 values actually decrease from the initial (here v2.1) to the latest (here v5.0) versions while the total gene counts increase. Nevertheless, assessments with the lower-resolution Metazoa lineage dataset (n=978) show parallel improvements in BUSCO completeness of both genomes and gene sets. When assessed with the high-resolution Aves lineage dataset (n=4,915), changes appear proportionally smaller but assembly completeness clearly improves. The latest official gene set (here v5.0) however appears to be not as good as the previous version (v4.0), as there are now slightly more missing BUSCOs, as well as more fragmented and duplicated BUSCOs.

Gene predictor training sets

Accurate gene model prediction is a complex task, especially when supporting evidence (e.g. native transcripts or homologs from other species aligned to the assembly) is lacking and predictions are performed *ab initio*. Gene prediction tools such as Augustus (Keller et al. 2011), GENEID (Blanco et al. 2007), GeneMark (Borodovsky and Lomsadze 2011), and SNAP (Korf 2004) need to optimize their parameter configurations for each genome in order to achieve the best results. Optimization of most predictors is usually performed through training steps that use sets of high-quality gene model annotations. Such sets are usually derived from full-length mature messenger RNAs (sequenced from the same species as that of the genome to be annotated) that have been aligned to the assembly. Annotation pipelines that combine several different types of gene model predictions may also undertake training steps, e.g. MAKER (Campbell et al. 2014) can employ the outputs of preliminary annotation runs to automatically retrain and improve its gene prediction algorithm. BUSCOs, being generally widely- and well-conserved genes, offer ideal predefined sets for such training procedures, without the need to first perform RNA sequencing for building initial sets of high-quality gene models.

Examining the effects of using BUSCO-trained parameters on the quality of the resulting gene model annotations reveals improvements, several of which are rather substantial, over using parameters pre-trained on the closest available species (Figure 2 main text, and Figure S1). Each analysis included running Augustus *ab initio* gene prediction across the whole genome using (i) BUSCO-trained Augustus parameters, and (ii) parameters provided by Augustus for the tested species or the closest available species. The *ab initio* predicted gene models for each species were then compared to the latest Official Gene Set (OGS) annotations to quantify how well these *ab initio* genes matched those of each OGS.

Unmasked genome assemblies and gene annotations were retrieved from Ensembl's FTP server (Ensembl release 89, Ensembl Metazoa release 35, and Ensembl Plants release 35), corresponding to: *Oreochromis niloticus, Tetraodon nigroviridis, Daphnia pulex, Danaus plexippus, Bombus impatiens, Nasonia vitripennis, Arabidopsis thaliana,*

Oryza sativa, Solanum lycopersicum, Drosophila melanogaster, Strigamia maritima, and *Tribolium castaneum.*

BUSCO v3.0.0 was run on each genome assembly using default settings and datasets: actinopterygii_odb9 (tilapia and *Tetraodon* pufferfish), embryophyta_odb9 (thale cress, Asian rice and tomato), diptera_odb9 (fruit fly), hymenoptera_odb9 (bumblebee and *Nasonia* wasp), arthropoda_odb9 (*Daphnia* and centipede) and endopterygota_odb9 (monarch butterfly and *Tribolium* beetle). Augustus was run on each unmasked genome using the following species metaparameters provided through Augustus: zebrafish (tilapia and *Tetraodon* pufferfish), arabidopsis (thale cress), rice (Asian rice), tomato (tomato), fly (fruit fly, monarch butterfly, *Daphnia* and centipede), honeybee (bumblebee), tribolium (*Tribolium* beetle) and nasonia (*Nasonia*).

Additionally, one Augustus run was performed for each genome using the parameters generated by the BUSCO v3.0.0 runs described above. In total two sets of *ab initio* gene predictions were generated for each of the genomes, one using metaparameters generated by BUSCO v3.0.0 and another using parameters supplied by Augustus belonging to the same species as the genome (e.g. tomato for tomato) or in their absence the closest available relative (e.g. zebrafish parameters for *Tetraodon* pufferfish).

These predicted proteins were then aligned against the respective reference OGS annotations using BLASTp. For each predicted protein, a coverage score was computed based on how well each prediction aligned to the reference sequences, a coverage score of 100% meaning that every amino acid of a reference protein is found in the *ab initio* predicted protein with no insertions, deletions or substitutions. As BUSCO employs Augustus for performing gene predictions, running an assembly assessment automatically provides users with the Augustus parameter configurations trained on the first round 'Complete' BUSCO matches. BUSCO results also include general feature format (GFF) and GenBank-formatted gene models for all 'Complete' BUSCO matches, so users can employ these as inputs for training other gene predictors.

Comparative genomic analyses

The rapid growth in the numbers of available genome assemblies means that during the design stages of many comparative genomics studies researchers may be faced with difficult decisions regarding which representative strains or species to include. Total gene counts are not always a good proxy for completeness as selecting those with the highest numbers of genes does not guarantee the best quality: genomes with a large gene counts are not necessarily the most complete and those with fewer genes are not necessarily less complete (Waterhouse 2015).

The choice of representatives will almost certainly be influenced by considerations of taxonomic sampling, the extent and/or accuracy of functional annotations, the availability of functional genomics data pertinent to the analyses, or simply historical usage (e.g. previously designated reference strains/species). However, all else being equal, quantitative assessments with BUSCO offer logical and intuitive selection criteria to help focus on the most complete genomic resources available. For example, running BUSCO v1 assessments of 653 publically available *Streptomyces* genomes revealed that complete recovery of all 40 bacteria BUSCOs was only possible for 63% of the assemblies (Studholme 2016). Thus, researchers wishing to include only a handful of representative *Streptomyces* genomes in their analyses could use BUSCO completeness metrics in combination with other considerations (see above) to help select the best-quality input data.

Assessing available genome assemblies and annotations for 135 *Lactobacillus* (bacteria) strains or species with the 443 BUSCOs of the lactobacillales dataset and 35 *Aspergillus* (fungi) species with the 4,046 BUSCOs of the eurotiomycetes dataset, shows that there is substantial variation in the completeness of these genomic resources (Figure S2).

The 135 *Lactobacillus* genomes were retrieved from NCBI RefSeq FTP using a custom script to download all references and representative genomic files for the *Lactobacillus* genus (28th April 2017). 35 *Aspergillus* genomes were downloaded manually from the NCBI genome browsers (16th February 2017). The N50 and L50 values were computed with a custom script. The fungal gene counts were retrieved from the NCBI genome

browsers and the bacterial gene counts were extracted from the GFF files by filtering unique gene id (unique on pattern "Parent[=]*gene[0-9]*" or "GeneID:[0-9]*"). BUSCO v3.0.0 was run on all bacteria and fungi using the dataset lactobacillales_odb9 (2016-11-01) and eurotiomycetes_odb9 (2017-01-26), respectively.

Genome assembly completeness is compared here to assembly N50 (half the genome found in scaffolds of length N50 or longer), assembly L50 (the number of longest scaffolds that span half the genome), and the number of annotated protein-coding genes. These results show:

- Of the four *Lactobacillus* designated reference strains (RefSeq), only one achieves 100% completeness, the other three achieve good scores of above 97% but importantly several non-references (some with shorter N50s and higher L50s) display slightly better scores.
- The three reference *Aspergillus* strains differ markedly in their N50 values but all score above 97% completeness, nevertheless there are several non-references with good scores and good contiguity metrics.
- Selecting assemblies with the highest gene counts does not necessarily guarantee better coverage of the true gene content of the species: for both the bacteria and fungi there are several examples with many more predicted genes but with fewer complete BUSCOs than other representatives, shown in panels **c** and **f**.
- BUSCO scores may help the user to weigh up the other quality information - tagged as reference, N50, L50, and gene content - in order to make an informed decision, especially when other metrics are not in full agreement with each other.
- NB: In panel **f** there are 8 fungi without gene predictions that are therefore not shown, the GFF file of the outlier with only 706 genes does contain only 706 annotations and this is reported on the NCBI genome page, but the publication reported 13,000 genes.

Reliable phylogenomics markers

Estimating true phylogenetic relationships among organisms is a prerequisite to almost any evolutionary study and usually employs mathematical methods to infer evolutionary histories based on evidence in the form of features from extant species (Delsuc et al. 2005). As the numbers of such features increase through the availability of whole genome and/or transcriptome sequences, or targeted genomic regions of interest e.g. with BaitFisher (Mayer et al. 2016), phylogenetics scales up to become phylogenomics. Powerful as this scaling up may be, care must be taken to account for potentially confounding effects from missing data, variable sequence divergence rates or compositions, incomplete lineage sorting or introgression, etc. to reach a confident conclusion. Recent notable examples include extensive transcriptomics to increase species sampling to examine the evolution of insects (Misof et al. 2014; Peters et al. 2017) and spiders (Fernández et al. 2014), and whole genome sequencing to build a well-supported avian phylogeny (Jarvis et al. 2014) and explore gene flow in mosquitoes (Fontaine et al. 2015).

Being near-universal single-copy genes, BUSCOs represent predefined sets of reliable markers where assessments of genomes, annotated gene sets, and/or transcriptomes can identify shared subsets from different types of genomic data for comprehensive phylogenomics studies. For example, phylogenomic analyses confirming that odonates (dragonflies and damselflies) are a sister lineage to neopterans employed single-copy orthologs of banded demoiselle genes from representatives of seven other insect orders as well as BUSCO-identified orthologs from two additional damselfly species for which only transcriptome data were available (Ioannidis et al. 2017).

The analysis of seven annotated rodent genomes together with five rodent transcriptomes illustrates the use of BUSCO for phylogenomics studies (Figure 3, main text). The BUSCO assessments identify conserved single-copy marker genes/proteins that are then used as input data for building a superalignment from which to estimate the species phylogeny. Analyses with the higher-resolution lineage datasets take longer to run but they identify many more single-copy orthologs from the genomes and transcriptomes to use for inferring the species phylogeny.

Protein sequences from seven rodent official gene sets from reference or representative assemblies were retrieved from NCBI: mouse, *Mus musculus*, GRCm38.p5 (GCF_000001635); rabbit, *Oryctolagus cuniculus*, OryCun2.0 (GCF_000003625); guinea pig, *Cavia porcellus*, Cavpor3.0 (GCF_000151735), kangaroo rat, *Dipodomys ordii*, Dord_2.0 (GCF_000151885); naked mole-rat, *Heterocephalus glaber*, HetGla_female_1.0 (GCF_000247695); degu, *Octodon degus*, OctDeg1.0 (GCF_000260255), and jerboa, *Jaculus jaculus*, JacJac1.0 (GCF_000280705). For each species, transcript isoforms were filtered to keep only the longest protein-coding sequence for each gene according to the corresponding GFF entries. BUSCO v2.0.0 was run on each set in protein mode, with the option -c 12 (i.e. 12 cores), and using the datasets euarchontoglires_odb9 (2016-11-01), mammalia_odb9 (2016-10-21), and metazoa_odb9 (2016-10-21).

Five rodent transcriptomes were selected from various published sources: akodont, *Abrothrix longipilis* [PRJNA256304, GCIS01, (Valdez et al. 2015)]; groundhog, *Marmota monax* [PRJNA291589, GDKO01, (Fletcher et al. 2015)]; dwarf hamster, *Phodopus campbelli* [PRJNA306772, GEVA01, (Brekke et al. 2016)]; mashona mole-rat, *Fukomys darlingi* [PRJNA303968, GFAQ01, (Omerbašić et al. 2016)]; and beaver, *Castor canadensis* [PRJNA359140, GFFV01 and GFFW01 merged, (Lok et al. 2017)]. BUSCO v2.0.0 was run using the same parameters and datasets as for proteins, but using the transcriptome mode.

For the three taxonomic levels (euarchontoglires, mammalia, and metazoa), a custom script was used to extract the respective BUSCO genes present in all species in single-copy. These genes were individually aligned using MAFFT (v7.305, options --thread -12 --auto) (Katoh and Standley 2013) and the alignments were filtered with trimAl (v1.4 rev10, option -automated1) (Capella-Gutiérrez et al. 2009). All alignments were concatenated and the maximum likelihood tree was produced using RAxML (v8.1.2, raxmlHPC-PTHREADS-SSE3 -T 12 -f a -m PROTGAMMAJTT -N 100 -n rodents -s $wd/supermatrix.aln.faa -p 13432 -x 89090) (Stamatakis 2014). The resulting tree was rooted with newick utilities (v1.6) (Junier and Zdobnov 2010). Scripts to reproduce this analysis can be downloaded from https://gitlab.com/ezlab/busco_usecases.

Assessments with the high-resolution Euarchontoglires lineage dataset identified 544 complete BUSCOs found in all species compared with 477 for the lower-resolution Mammalia dataset and just 155 for the low-resolution Metazoa dataset (Figure 3, main text). The average BUSCO runtime for each species is clearly much shorter when using the low-resolution Metazoa dataset, but the higher resolution datasets identify more markers for downstream analysis.

In each case, the superalignment of these BUSCOs used for phylogenetic inference produces a maximum likelihood phylogeny that agrees with previously published results (Huchon et al. 2007) and (Blanga-Kanfi et al. 2009). The BUSCO-based tree topology fully matches the trees presented in Huchon *et al.* for all species in common. Blanga-Kanfi *et al.* contains a family absent from Huchon *et al.* (Cricetidae, represented by *Microtus* and *Mesocricetus,* to which *Phodopus* and *Abrothrix* belong), in agreement with the BUSCO-based phylogeny, but disagrees with Huchon *et al.* and thus the BUSCO-based phylogeny on where to position *Marmota*, explainable by a very short branch leading to the node.

In this analysis we deliberately did not pre-filter transcriptomes to maintain only one transcript per gene, this often leads to large proportions of discarded duplicate BUSCOs as seen on Figure 3 (main text), but it demonstrates how a large clade-specific BUSCO dataset can identify enough genes from genomic data of variable quality, thus avoiding the burden of manual and possibly biased selection of orthologs. This example illustrates the utility of BUSCO assessments to relatively quickly and easily identify reliable single-copy markers from different types of genomic data for use in large-scale phylogenomics studies.

The rodent example above used BUSCO in protein mode on the annotated gene sets and in transcriptome mode on the species for which only transcripts were available. Using BUSCO in genome mode is also a useful approach to identify universal single-copy markers, e.g. to reconstruct the yeast phylogeny of Saccharomycotina from available genome data, BUSCO assessments of 96 genomes identified more than 1,000 markers for phylogenomic inference (Shen et al. 2016).

Supplementary References

Blanco E, Parra G, Guigó R. 2007. Using geneid to Identify Genes. Curr. Protoc. Bioinforma. Chapter 4:Unit 4.3.

Blanga-Kanfi S, Miranda H, Penn O, Pupko T, DeBry RW, Huchon D. 2009. Rodent phylogeny revised: analysis of six nuclear genes from all major rodent clades. BMC Evol. Biol. 9:71.

Borodovsky M, Lomsadze A. 2011. Eukaryotic gene prediction using GeneMark.hmm-E and GeneMark-ES. Curr. Protoc. Bioinforma. Chapter 4:Unit 4.6.1-10.

Brekke TD, Henry LA, Good JM. 2016. Genomic imprinting, disrupted placental expression, and speciation. Evolution 70:2690–2703.

Camacho C, Coulouris G, Avagyan V, Ma N, Papadopoulos J, Bealer K, Madden TL. 2009. BLAST+: architecture and applications. BMC Bioinformatics 10:421.

Campbell MS, Holt C, Moore B, Yandell M. 2014. Genome Annotation and Curation Using MAKER and MAKER-P. Curr. Protoc. Bioinforma. 48:4.11.1-39.

Capella-Gutiérrez S, Silla-Martínez JM, Gabaldón T. 2009. trimAl: a tool for automated alignment trimming in large-scale phylogenetic analyses. Bioinformatics 25:1972–1973.

Chin C-S, Peluso P, Sedlazeck FJ, Nattestad M, Concepcion GT, Clum A, Dunn C, O'Malley R, Figueroa-Balderas R, Morales-Cruz A, et al. 2016. Phased diploid genome assembly with single-molecule real-time sequencing. Nat. Methods 13:1050–1054.

Davey JW, Chouteau M, Barker SL, Maroja L, Baxter SW, Simpson F, Joron M, Mallet J, Dasmahapatra KK, Jiggins CD. 2016. Major Improvements to the Heliconius melpomene Genome Assembly Used to Confirm 10 Chromosome Fusion Events in 6 Million Years of Butterfly Evolution. G3 (Bethesda). 6:695–708.

Delsuc F, Brinkmann H, Philippe H. 2005. Phylogenomics and the reconstruction of the tree of life. Nat. Rev. Genet. 6:361–375.

Eddy SR. 2011. Accelerated Profile HMM Searches. PLoS Comput Biol 7:e1002195.

Elsik CG, Worley KC, Bennett AK, Beye M, Camara F, Childers CP, de Graaf DC, Debyser G, Deng J, Devreese B, et al. 2014. Finding the missing honey bee genes: lessons learned from a genome upgrade. BMC Genomics 15:1–29.

Fernández R, Hormiga G, Giribet G. 2014. Phylogenomic analysis of spiders reveals nonmonophyly of orb weavers. Curr. Biol. 24:1772–1777.

Fletcher SP, Chin DJ, Gruenbaum L, Bitter H, Rasmussen E, Ravindran P, Swinney DC, Birzele F, Schmucki R, Lorenz SH, et al. 2015. Intrahepatic Transcriptional Signature Associated with Response to Interferon-α Treatment in the Woodchuck Model of Chronic Hepatitis B.Robek MD, editor. PLoS Pathog. 11:e1005103.

Fontaine MC, Pease JB, Steele A, Waterhouse RM, Neafsey DE, Sharakhov I V, Jiang X, Hall

AB, Catteruccia F, Kakani E, et al. 2015. Extensive introgression in a malaria vector species complex revealed by phylogenomics. Science (80-.). 347:1258524–1258524.

Huchon D, Chevret P, Jordan U, Kilpatrick CW, Ranwez V, Jenkins PD, Brosius J, Schmitz J. 2007. Multiple molecular evidences for a living mammalian fossil. Proc. Natl. Acad. Sci. U. S. A. 104:7495–7499.

Ioannidis P, Simao FA, Waterhouse RM, Manni M, Seppey M, Robertson HM, Misof B, Niehuis O, Zdobnov EM. 2017. Genomic features of the damselfly Calopteryx splendens representing a sister clade to most insect orders. Genome Biol. Evol. 9:415–430.

Jarvis ED, Mirarab S, Aberer AJ, Li B, Houde P, Li C, Ho SYW, Faircloth BC, Nabholz B, Howard JT, et al. 2014. Whole-genome analyses resolve early branches in the tree of life of modern birds. Science 346:1320–1331.

Junier T, Zdobnov EM. 2010. The Newick utilities: high-throughput phylogenetic tree processing in the Unix shell. Bioinformatics 26:1669–1670.

Katoh K, Standley DM. 2013. MAFFT multiple sequence alignment software version 7: improvements in performance and usability. Mol. Biol. Evol. 30:772–780.

Keller O, Kollmar M, Stanke M, Waack S. 2011. A novel hybrid gene prediction method employing protein multiple sequence alignments. Bioinformatics 27:757–763.

Korf I. 2004. Gene finding in novel genomes. BMC Bioinformatics 5:59.

Lok S, Paton TA, Wang Z, Kaur G, Walker S, Yuen RKC, Sung WWL, Whitney J, Buchanan JA, Trost B, et al. 2017. De Novo Genome and Transcriptome Assembly of the Canadian Beaver (Castor canadensis). G3 (Bethesda). 7:755–773.

Mayer C, Sann M, Donath A, Meixner M, Podsiadlowski L, Peters RS, Petersen M, Meusemann K, Liere K, Wägele J-W, et al. 2016. BaitFisher: A Software Package for Multispecies Target DNA Enrichment Probe Design. Mol. Biol. Evol. 33:1875–1886.

Misof B, Liu S, Meusemann K, Peters RS, Donath A, Mayer C, Frandsen PB, Ware J, Flouri T, Beutel RG, et al. 2014. Phylogenomics resolves the timing and pattern of insect evolution. Science (80-.). 346:763–767.

Omerbašić D, Smith ESJ, Moroni M, Homfeld J, Eigenbrod O, Bennett NC, Reznick J, Faulkes CG, Selbach M, Lewin GR. 2016. Hypofunctional TrkA Accounts for the Absence of Pain Sensitization in the African Naked Mole-Rat. Cell Rep. 17:748–758.

Parra G, Bradnam K, Korf I. 2007. CEGMA: a pipeline to accurately annotate core genes in eukaryotic genomes. Bioinformatics 23:1061–1067.

Peters RS, Krogmann L, Mayer C, Donath A, Gunkel S, Meusemann K, Kozlov A, Podsiadlowski L, Petersen M, Lanfear R, et al. 2017. Evolutionary History of the Hymenoptera. Curr. Biol. 27:1013–1018.

Shen X-X, Zhou X, Kominek J, Kurtzman CP, Hittinger CT, Rokas A. 2016. Reconstructing the Backbone of the Saccharomycotina Yeast Phylogeny Using Genome-Scale Data. G3

Genes|Genomes|Genetics 6.

Simão FA, Waterhouse RM, Ioannidis P, Kriventseva E V., Zdobnov EM. 2015. BUSCO: Assessing genome assembly and annotation completeness with single-copy orthologs. Bioinformatics 31:3210–3212.

Stamatakis A. 2014. RAxML version 8: a tool for phylogenetic analysis and post-analysis of large phylogenies. Bioinformatics 30:1312–1313.

Studholme DJ. 2016. Genome Update. Let the consumer beware: *Streptomyces* genome sequence quality. Microb. Biotechnol. 9:3–7.

Tørresen OK, Star B, Jentoft S, Reinar WB, Grove H, Miller JR, Walenz BP, Knight J, Ekholm JM, Peluso P, et al. 2017. An improved genome assembly uncovers prolific tandem repeats in Atlantic cod. BMC Genomics 18:95.

Valdez L, Giorello F, Feijoo M, Opazo JC, Lessa EP, Naya DE, D'Elía G. 2015. Characterization of the kidney transcriptome of the long-haired mouse Abrothrix hirta (Rodentia, Sigmodontinae) and comparison with that of the olive mouse A. olivacea.Armando I, editor. PLoS One 10:e0121148.

Warren WC, Hillier LW, Tomlinson C, Minx P, Kremitzki M, Graves T, Markovic C, Bouk N, Pruitt KD, Thibaud-Nissen F, et al. 2017. A New Chicken Genome Assembly Provides Insight into Avian Genome Structure. G3 (Bethesda). 7:109–117.

Waterhouse RM. 2015. A maturing understanding of the composition of the insect gene repertoire. Curr. Opin. Insect Sci. 7:15–23.

Waterhouse RM, Tegenfeldt F, Li J, Zdobnov EM, Kriventseva E V. 2013. OrthoDB: A hierarchical catalog of animal, fungal and bacterial orthologs. Nucleic Acids Res. 41:D358-65.

Waterhouse RM, Zdobnov EM, Kriventseva E V. 2011. Correlating traits of gene retention, sequence divergence, duplicability and essentiality in vertebrates, arthropods, and fungi. Genome Biol. Evol. 3:75–86.

Wickham H. 2009. ggplot2: Elegant Graphics for Data Analysis, http://ggplot2.org. Springer-Verlag New York.

Zdobnov EM, Tegenfeldt F, Kuznetsov D, Waterhouse RM, Simao FA, Ioannidis P, Seppey M, Loetscher A, Kriventseva E V. 2017. OrthoDB v9.1: Cataloging evolutionary and functional annotations for animal, fungal, plant, archaeal, bacterial and viral orthologs. Nucleic Acids Res. 45:D744–D749.

Supplementary Figures

Figure S1. BUSCO-trained *ab initio* gene prediction with Augustus. Examining the effects of using BUSCO-trained parameters on the quality of the resulting gene model annotations reveals improvements, several of which are rather substantial, over using parameters pre-trained on the closest available species. Each analysis included running Augustus *ab initio* gene prediction using (i) BUSCO-trained parameters, and (ii) parameters provided by Augustus for the tested species or the closest available species. The *ab initio* gene models for each species were then compared to the Official Gene Set (OGS) to quantify how well these genes matched those of each OGS.

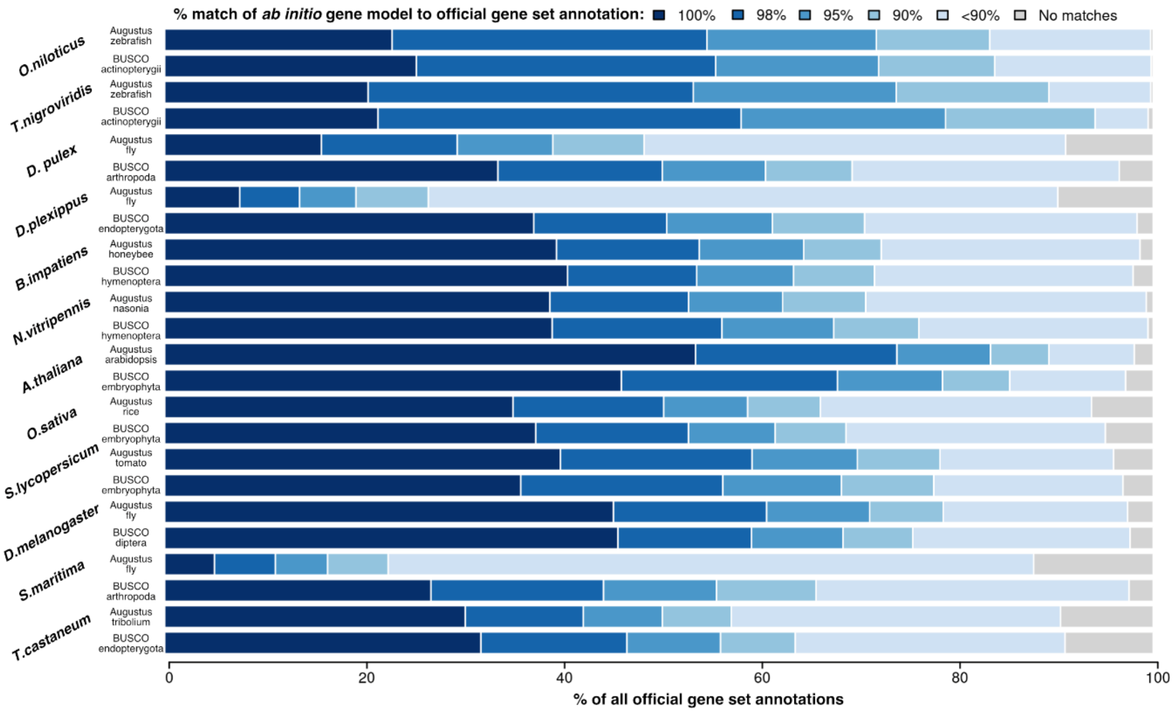

Figure S2. Comparisons of contiguity metrics (**a, b, d, e**) and gene counts (**c, f**) versus BUSCO completeness scores for 135 *Lactobacillus* (**a-c**) and 35 *Aspergillus* (**d-f**) genome assemblies. The four bacterial and three fungal RefSeq designated reference species/strains are represented by different coloured triangles. The most specific available BUSCO dataset was used for each clade: lactobacillales (n=443) and eurotiomycetes (n=4,046).

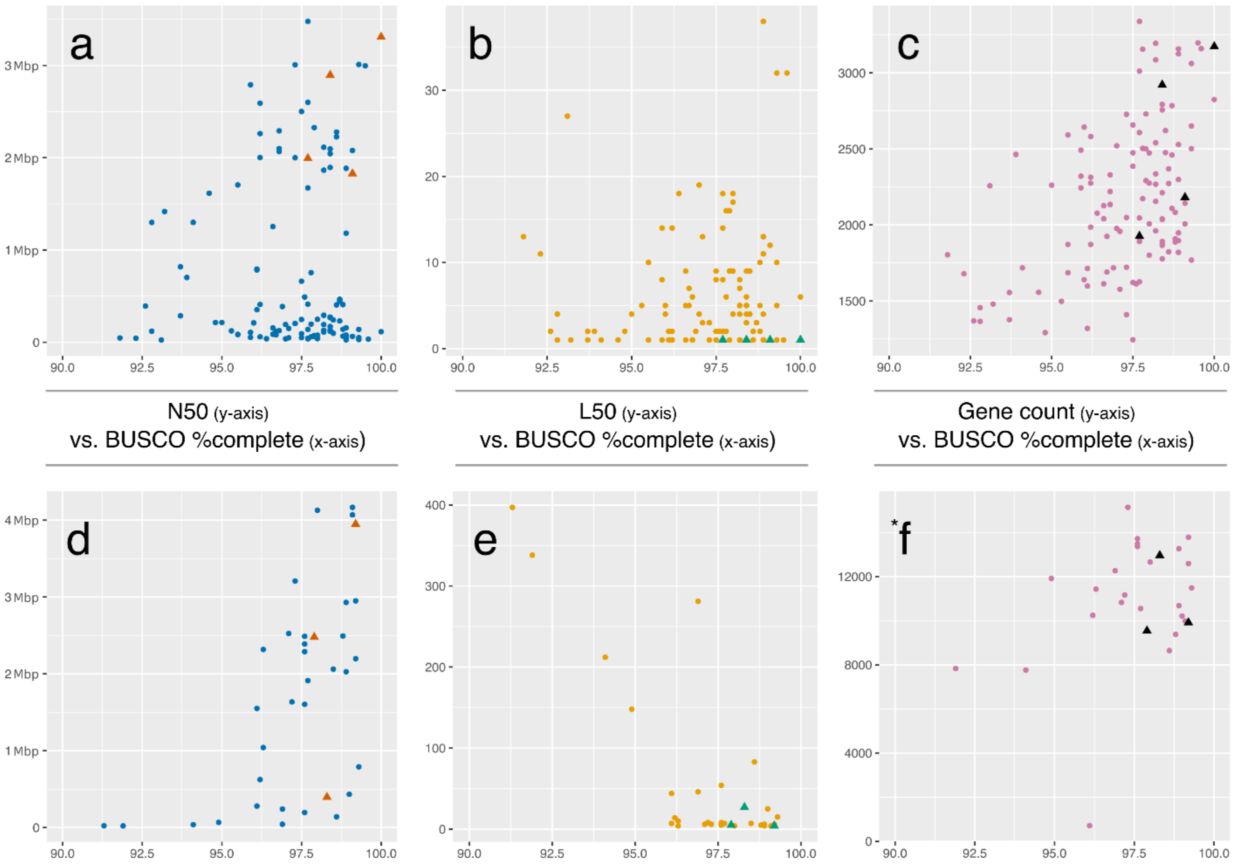

Supplement: Supplementary Data [file msx319_supp.pdf]
